# Gender differences in willingness for childbirth, fertility knowledge, and value of motherhood or fatherhood and their associations among college students in South Korea, 2021

**DOI:** 10.1186/s13690-023-01127-x

**Published:** 2023-06-16

**Authors:** Hae Won Kim, Seo Yun Kim

**Affiliations:** 1 Research Institute of Nursing Science, Center for Human-Caring Nurse Leaders for the Future by Brain Korea 21 (BK 21) Four Project, College of Nursing, Seoul National University, Seoul, Republic of Korea; 2 Nell Hodgson Woodruff School of Nursing, Emory University, Atlanta, USA

**Keywords:** Fertility, Intention, Knowledge, Parenthood, Gender, Preconception Care

## Abstract

**Background:**

South Korea is a country with a very low fertility rate and there is a tendency for young adults to postpone marriage and childbirth, which affects adverse pregnancy outcomes. It is important for young adults to predict and prepare in advance for future fertility-related issues, especially by identifying their willingness and thoughts about childbirth for both women and men. This study aimed to investigate gender differences in willingness for childbirth, fertility knowledge, and the value of motherhood or fatherhood among college students in South Korea and to explore the factors influencing willingness for childbirth.

**Methods:**

This was a cross sectional study among 286 unmarried college students who were recruited through a campus email and online communities for college student conducted from June 20, 2021 to July 19, 2021. The data were analyzed using the chi-square test and t-test to identify gender differences in general characteristics, willingness for childbirth, fertility knowledge, and value of motherhood of fatherhood. The factors influencing willingness for childbirth were examined using multiple logistic regression.

**Results:**

Female students showed lower willingness for future childbirth than male students (χ^2^ = 26.85, *p* < .001). Women valued motherhood significantly less than men valued fatherhood (t = 6.34, *p* < .001). Women had a lower fertility knowledge score than men (t = 2.53 *p* = .012). The value of motherhood or fatherhood was an important influencing factor in both male and female college students (AOR = 8.57, 95% CI = 3.79–19.41 and AOR = 10.42, 95% CI = 3.65–29.80, respectively), whereas the monthly allowance was also an important influencing factor in female students (AOR = 1.02, 95% CI = 1.01–1.03).

**Conclusion:**

The findings indicate the future direction to develop effective interventions considering gender differences which will promote healthy pregnancy and childbirth in the future for informed reproductive decision-making for college students.

**Table:** 

Text box 1: Contributions to the literature	
•The Study findings make a significant contribution by focusing on gender differences in the future childbirth willingness, fertility knowledge, and value of motherhood or fatherhood of college students as well as by identifying significant influencing factors for future childbirth willingness.	
•This study is significant because investigating college students’ willingness and thoughts about future parenthood provides an opportunity to explore planning for childbearing in advance, which might improve healthy pregnancy and childbirth in the future.	
•These findings could inform the development of effective interventions for college students to promote fertility and healthy pregnancy and childbirth in the future considering gender differences.	

## Introduction

South Korea (hereafter, Korea) has a very low fertility rate [1], which is the lowest among Organization for Economic Co-operation and Development countries [2]. Moreover, young people are more likely to delay marriage or having children, increasing the proportion of mothers with advanced maternal age, which is associated with higher risks of adverse pregnancy outcomes [3–5]. The Centers for Disease Control and Prevention recommend the concept of reproductive life planning (RLP), in which people think about their desires to have or not have children and set goals in the context of individual values across a person’s life span [6]. From the perspective of RLP, for unmarried young people, including college students who are likely to get married, become pregnant, or have children in the near future, it is important to anticipate and prepare in advance for future fertility-related problems, especially by examining their willingness and thoughts about childbirth. Both young women and men should engage in this process. Furthermore, it is suggested in previous studies that it is necessary to evaluate fertility intention and awareness and provide education for college students to assist them in making more informed reproductive decisions [7]. Additionally, since college students are becoming more sexually active and entering adulthood, assessing their willingness and thoughts about childbirth, and educating them on planning healthy pregnancies in the future will be necessary.

Several studies have investigated awareness of future childbirth, fertility, and attitudes toward childbirth among unmarried young people; in particular, differences between men and women are evident. According to a study on Korean university students, male students were more likely to be willing to have children in the future than female students, and they tended to think that having a child would be important in their lives [7, 8]. Moreover, men had a higher level of fertility awareness than women [7–9] and had more positive attitudes toward childbearing [7]. However, other studies from the United States and Denmark showed opposite trends [10, 11]. Female students were more likely than male students to be willing to have children and to consider having children important, even though fertility awareness was similarly low in both men and women [10, 11].

In a previous study, fertility knowledge was associated with willingness for future marriage and childbirth; specifically, women who expressed willingness for marriage and childbirth had more fertility knowledge than women who did not among college students [8]. In addition, higher values placed on marriage, childbirth, and children were associated with stronger intentions to have children [7, 12, 13]. Furthermore, younger age and lower socioeconomic status were associated with less willingness for childbirth [14]. Willingness for childbirth and childbearing intention have also been found to be negatively associated with unhealthy behaviors such as drinking and smoking [15]. Sexual experience was also identified as a significant predictor of fertility intention in young women [16]. In light of these results, it is necessary to identify factors affecting willingness for future childbearing among college students, including these variables.

In summary, this study investigated the willingness for childbirth in the future and related thoughts according to gender among college students, who are likely to become parents in the near future, and explored the factors affecting willingness for future childbirth. This information could be used to verify potentially modifiable individual factors and inform the development of effective interventions to promote fertility and healthy pregnancy and childbirth.

The purpose of this study was to understand gender differences in willingness for childbirth, fertility knowledge, and the value of motherhood or fatherhood among college students in Korea and to explore their associations. In detail, the goals of this study were (1) to identify gender differences in general characteristics, willingness for childbirth, fertility knowledge, and value of motherhood or fatherhood; (2) to investigate factors associated with willingness for childbirth by gender; and (3) to explore the factors influencing willingness for childbirth by gender among college students.

## Methods and materials

### Study design, setting, and participants

We conducted a cross-sectional survey using an online questionnaire. The inclusion criteria were college students who were unmarried and aged 18 or older in Korea. The participants of this study were college students simultaneously recruited (1) through S university campus e-mails in Korea, and (2) through the posted participant recruitment documents on the two most popular online communities for college students on one of Korean famous web portal (Nav**). The subjects could participate in the study through the URL linked in the recruitment documents or in the e-mails. The sample size for logistic regression was calculated using G*Power version 3.1.9.2. For sample size calculation, we used 5% alpha, 80% power, and an odds ratio (OR) of 1.5 (moderate effect size); as a result, the required sample size was 242. Considering the dropout rate of 25% described in a previous study conducted using an online survey through an online community for college students [17], the final number of students required was estimated to be 305.

In total, 303 college students participated in the study. After excluding responses for which more than 10% of all items had incomplete responses, the final analyzed sample consisted of 286 students (144 men, 142 women).

### Measures

The questionnaire contained items on the following parameters: general characteristics of subjects (sociodemographic factors and health behavior characteristics), willingness for childbirth (including marriage), fertility knowledge, and value of motherhood or fatherhood.

### General characteristics

The questionnaire consisted of items related to sociodemographic characteristics, including age in years, gender, major (health-related/non-health-related), religion (yes; Christianity, Catholicism, Buddhism, others/no), allowance per month (including both pocket money and part-time job salary), and residence type (parent’s home/self-boarding/dormitory/other) and health behavior characteristics, including smoking (not at all/past only/currently), alcohol drinking frequency (not at all or less than one time per month/2–4 times per month/more than 2 times per week), experience of sexual intercourse (not at all/past only/currently [in the previous month]), and contraception (never/sometimes/often/always), and experience of pregnancy (yes/no).

### Willingness for childbirth (including marriage)

Willingness for childbirth and marriage included 5-items on willingness for future childbirth (yes/no/unsure), expected childbirth age (open response format), desired number of children (open response format), willingness for future marriage (yes/no/unsure), expected marriage age childbirth (open response format).

### Fertility knowledge

Fertility knowledge was measured through the tool developed by Bunting et al. [18], the construct validity of which was confirmed among college students in Korea [9].

The scale consists of 13-items that assess indicators of reduced fertility, misconceptions about fertility, and basic facts about infertility. Each item is answered “true,” “false,” or “don’t know.” A correct answer is scored 1 point and an incorrect answer or “don’t know” is given 0 points. The range of scores is 0 to 13, and a high score indicates a high knowledge level.

The reliability of the fertility knowledge scale was confirmed by Cronbach’s alpha values of 0.79 [18] and 0.60 [9] in the previous study; Cronbach’s alpha value was 0.63 in the current study.

### Value of motherhood or fatherhood

The instrument for the value of motherhood was developed by the researcher in a previous study [19]. We modified two items for the value of fatherhood based on the instrument for the value of motherhood. The scale consists of eight items that assess the value of pregnancy, childbirth, and the role of mother or father. The responses to each item are scored using a 4-point Likert scale and the range of scores is 8 to 32. Higher scores reflect a higher value of motherhood or fatherhood. Cronbach’s alpha for the instrument dealing with the value of motherhood was 0.56 in the previous study [19], and the present study found Cronbach’s alpha values of 0.72 for the value of fatherhood among male students and 0.76 for the value of motherhood among female students.

### Data collection

This study was approved by the Institutional Review Board (IRB) of S university (No. 2103/003–004) in Korea. The survey was conducted from June 20, 2021 to July 19, 2021. Each student in this study was sent an e-mail or completed a posted recruitment document from the online community website containing a description of the research with a link to the questionnaire via SurveyMonkey (an online survey website). Before starting the survey, prospective participants were informed that they could voluntarily participate in the study and withdraw their decision at any time on the web screen, and only those who agreed to do so proceeded to participate in the study. After the study was completed, an e-gift (worth KRW 4,000 or approximately USD 3.50) was provided to all participants as a token of appreciation for participation in the study.

### Data analysis

Statistical analyses were performed using SPSS version 24 (IBM Corp., Armonk, NY, USA). The chi-square test and the independent t-test were utilized to analyze differences in general characteristics (socio-demographic characteristics and health behavior characteristics), willingness for childbirth (including marriage), fertility knowledge, and value of motherhood of fatherhood between male and female college students. The chi-square test was used to analyze differences in socio-demographic and health behavior characteristics and fertility knowledge according to willingness for childbirth between men and women. The factors influencing willingness for childbirth were examined through multiple logistic regression. The independent variables were fertility knowledge converted to a dichotomous variable according to the mean score for total knowledge (scores of 0–6 vs. 7–13 for both male and female students) and value of motherhood of fatherhood converted to a dichotomous variable according to the mean total scores (8–25 vs. 26–32 for male students and 8–22 vs. 23–32 for female students). The dependent variable (willingness for childbirth) was categorized as willingness for childbirth (responses of “yes”) and unwillingness or uncertainty about childbirth (responses of “not willing” and “don’t know”).

## Results

### Comparison of general characteristics between men and women

The participants included 286 college students, of whom 144 were men and 142 were women. The mean age was 22.19 ± 2.41 years (range, 18–35 years) in the overall participants (22.92 ± 2.50 years for men and 21.45 ± 2.08 years for women). Most respondents majored in non-health-related subjects (82.5%) and were not religious (75.2%). Their mean monthly allowance was KRW 49.66 ± 34.98 (× 10,000) (e.g. approximately USD 400) and 55.6% reported that they lived with their parents. Most participants (80.1%) had not smoked in the past or currently, 90.2% had engaged in alcohol drinking, and 47.9% had sexual experience (among whom 24.1% did not always use contraception).

The socio-demographic characteristics and health behavior characteristics that showed a statistically significant difference according to gender were age (t = 5.40, *p* < .001), smoking (χ^2^ = 24.91, *p* < .001), alcohol drinking (χ^2^ = 18.95, *p* = .002), sexual experience (χ^2^ = 16.38, *p* < .001), and contraception use (χ^2^ = 18.14, *p* = .001) (Table 1).

**Table 1:** Comparisons of general characteristics between men and women among college students from South Korea in 2021 (N = 286)

Variables		Categories	Total		Men (n = 144)		Women (n = 142)		χ^2^ or t(*p*)	
			n(%) or M ± SD							
Socio-demographic characteristics										
Age (years)		Range (18 ~ 35)	22.19 ± 2.41		22.92 ± 2.50		21.45 ± 2.08		5.40(< 0.001)	
		18–20	80(28.0)		31(21.5)		49(34.5)		9.05(0.029)	
		21–25	188(65.7)		100(69.4)		88(62.0)			
		26–35	16(5.6)		13(7.6)		5(3.5)			
Major category		Non-health-related	236(82.5)		121(84.7)		115(81.0)		0.46(0.498)	
		Health-related	50(17.5)		23(16.0)		27(19.0)			
Religious		No	215(75.2)		114(79.2)		101(75.2)		2.48(0.116)	
		Yes	71(24.8)		30(20.8)		41(28.9)			
Allowance (10,000 won)		Range (0 ~ 300)	49.66 ± 34.98		47.97 ± 30.62		51.39 ± 38.93		-0.83(0.409)	
Residence type		Parent’s home	159(55.6)		72(50)		87(61.3)		6.05(0.109)	
		Self-boarding	70(24.5)		44(30.6)		26(18.3)			
		Dormitory	53(18.5)		26(18.1)		27(19.0)			
		Other	4(1.4)		2(1.4)		2(1.4)			
**Health behavior characteristics**										
Smoking frequency		Not at all	229(80.1)		99(68.8)		130(91.5)		24.91 (< 0.001)	
		Past only	24(8.4)		18(12.5)		6(4.2)			
		Currently	33(11.5)		27(18.8)		6(4.2)			
Alcohol drinking (times/ month or week)		Not at all ≤ 1/month 2–4/month ≥ 2/week	28(9.8) 129(45.1) 90(31.5) 39(13.6)		8(14.1) 57(39.6) 51(35.4) 28(19.5)		20(13.9) 72(51.6) 39(27.5) 11(7.7)		18.95(0.002)	
Sexual experience		Not at all Past only Currently	149(52.1) 75(26.2) 62(21.7)		58(40.3) 46(31.9) 40(27.8)		91(64.1) 29(20.4) 22(15.5)		16.38(< 0.001)	
Contraception use (n = 137)		Never Sometimes Often Always	5(3.6) 8(5.8) 20(14.6) 104(75.9)		2(2.3) 4(4.7) 12(14.0) 68(79.1)		3(5.9) 4(7.8) 8(15.7) 36(70.6)		18.14(0.001)	

*M* Mean; *SD* Standard deviation

### Comparison of willingness for childbirth, fertility knowledge, and value of motherhood or fatherhood between men and women

Male students showed more willingness for future childbirth than female students (χ^2^ = 26.85, *p* < .001). A statistically significant difference was found between men and women according to expected age at childbirth (t = 3.06, *p* < .003), and expected age at marriage (t = 2.23 *p* = .027). However, there was no significant difference in the desired number of children between both groups (Total 2.10 ± 0.56 children, 2.16 ± 0.57 children for men, and 2.00 ± 0.53 children for women). Male students were also more likely to express willingness for future marriage than female students (χ^2^ = 14.68, *p* = .001).

The mean score of men for fertility knowledge was significantly higher than that of women (t = 2.53 *p* = .012). Furthermore, men valued fatherhood significantly more than women valued motherhood (t = 6.34, *p* < .001) (Table 2).

**Table 2:** **Comparisons of willingness for childbirth, fertility knowledge, and value of motherhood or fatherhood between men and women among college students from South Korea in 2021 (N = 286)**

	Variables	Categories	Total	Men (n = 144)	Women (n = 142)	χ^2^ or t(*p*)
			n(%) or M ± SD			
Willingness for childbirth (including marriage)						
	Willingness for future childbirth	No	68(23.8)	17(11.8)	51(35.9)	26.85 (< 0.001)
		Yes	139(48.6)	88(61.1)	51(35.9)	
		Unsure	79(27.6)	39(27.1)	40(28.2)	
	Expected childbirth age (n = 139)	Range (28–39)	32.32 ± 2.32	32.76 ± 2.25	31.55 ± 2.27	3.06 (0.003)
		28–30	29(20.9)	12(13.6)	17(33.3)	9.04 (0.011)
		31–34	82(59.0)	54(61.4)	28(54.9)	
		≥ 35	28(20.1)	22(25.0)	6(11.8)	
	Desired number of children (n = 139)	Range (1–4)	2.10 ± 0.56	2.16 ± 0.57	2.00 ± 0.53	1.68 (0.096)
		1	12(4.2)	6(6.9)	6(11.8)	3.32 (0.190)
		2	103(36.0)	63(72.4)	40(78.4)	
		≥ 3	23(20.7)	18(8.4)	5(9.8)	
	Willingness for future marriage	No	32 (11.2)	7(4.9)	25(17.6)	14.68 (0.001)
		Yes	195(68.2)	111(77.1)	84(59.2)	
		Unsure	59(20.6)	26(18.1)	33(23.2)	
	Expected marriage age (n = 195)	Range (24–50)	30.90 ± 2.87	31.30 ± 2.36	30.38 ± 3.37	2.23 (0.027)
		24–27	14(7.2)	4(3.6)	10(11.9)	12.26 (0.007)
		28–30	93(47.7)	46(41.4)	47(56.0)	
		31–34	73(37.4)	51(45.9)	22(26.2)	
		≥ 35	15(7.7)	10(9.0)	5(6.0)	
**Fertility knowledge**		Range (0–13)	7.40 ± 2.12	7.72 ± 2.11	7.08 ± 2.09	2.53 (0.012)
**Value of motherhood or fatherhood**		Range (8–32)	25.0 ± 4.24	26.51 ± 3.68	23.54 ± 4.25	6.34 (< 0.001)

*M* Mean; *SD* Standard deviation

### Factors associated with willingness for childbirth between men and women

Upon cross-analyzing the responses on willingness for childbirth according to general characteristics, fertility knowledge, and value of motherhood or fatherhood, the variables that showed statistically significant differences according to willingness for childbirth were religion (χ^2^ = 3.86, *p* = .049), and the value of fatherhood (χ^2^ = 32.85, *p* < .001) for men, whereas the corresponding variables for women were religion (χ^2^ = 4.15, *p* = .042), allowance (χ^2^ = 2.12, *p* = .036), and the value of motherhood (χ^2^ = 19.95, *p* < .001) (Table 3).

**Table 3:** Comparisons of general characteristics, fertility knowledge, and value of motherhood or fatherhood by willingness for childbirth between men and women among college students from South Korea in 2021 (N = 286)

Variables	Categories	Total	Men (n = 144)					Women (n = 142)		
			Willingness for childbirth (n = 88)	Unwillingness or uncertainty about childbirth (n = 56)		χ^2^ (*p*) or t (*p*)		Willingness for childbirth (n = 51)	Unwillingness or uncertainty about childbirth (n = 91)	χ^2^ (*p*) or t (*p*)
		n(%) or M ± SD						n(%) or M ± SD		
**Socio-demographic characteristics**										
Age		22.19 ± 2.41	22.80 ± 2.73		23.11 ± 2.09	-0.73 (0.467)		21.80 ± 2.00	21.25 ± 2.10	1.53 (0.129)
Major category	Non-health-related	236(82.5)	72(50.0)		49(34.0)	0.82 (0.364)		41(28.9)	74(52.1)	0.02 (0.893)
	Health-related	50(17.5)	16(11.1)		7(4.9)			10(7.0)	17(12.0)	
Religious	No	215(75.2) 71(24.8)	65(45.1) 23(16.0)		49(34.0) 7(4.9)	3.86 (0.049)		31(21.8)	70(49.3)	4.15 (0.042)
	Yes							20(14.1)	21(14.8)	
Allowance (won/month)		49.66 ± 34.98	51.32 ± 30.42		42.70 ± 30.45	1.66 (0.100)		60.53 ± 40.75	46.26 ± 3.89	2.12 (0.036)
Residence type	Parent’s home	159(55.6)	44(30.6)		28(19.4)	0.00 (1.000)		29(20.4)	58(40.8)	0.65 (0.420)
	Self-boarding, dormitory, and other	127(44.4)	44(30.6)		28(19.4)			22(15.5)	33(23.2)	
**Health behavior characteristic**										
Smoking	Yes (past or current)	57(19.9)	28(19.4)		17(11.8)	0.03 (0.854)		5(3.5)	7(4.9)	0.19 (0.664)
	No	229(80.1)	60(41.7)		39(27.1)			46(32.4)	84(59.2)	
Alcohol drinking	Yes (≥ 1)	258(90.2) 28(9.8)	85(59.0) 3(2.1)		51(35.4) 5(3.5)	1.99 (0.159)		44(31.0)	78(54.9)	0.01 (0.927)
	No							7(4.9)	13(9.2)	
Sexual experience	Yes (past or current)	137(47.9) 149(51.1)	56(38.9) 32(22.2)		30(20.8) 26(18.1)	1.44 (0.230)		20(14.1)	31(21.8)	0.38 (0.539)
	No							31(21.8)	60(42.3)	
Fertility knowledge	Low score (0–6)	98(34.3) 188(65.7)	39(27.1) 49(34.0)		31(21.5) 25(17.4)	1.67 (0.196)		29(20.4)	55(38.7)	0.17 (0.677)
	High score (7–13)							22(15.5)	36(25.4)	
Value of motherhood or fatherhood	Low score (8–25) ^a^ / (8–22) ^b^	106(37.1) 180(62.9)	13(9.0) 75(52.1)		34(23.6) 22(15.3)	32.85 (< 0.001)		7(4.9)	47(33.1)	19.95 (< 0.001)
	High score (26–32) ^a^ / (23–32) ^b^							44(31.0)	44(31.0)	

^a^ Based on mean scores of total values for men; ^b^ Based on mean scores of total values for women; *M* Mean; *SD* Standard deviation

In terms of health behavior, no significant difference was found regarding smoking, alcohol drinking, and sexual experience by willingness for childbirth in either men or women. There was no significant difference in fertility knowledge according to willingness for childbirth in the future in either gender group.

### Factors influencing willingness for childbirth in men and women

In the analysis of factors influencing willingness for childbirth in the future, a higher value of fatherhood was associated with a higher likelihood of willingness for childbirth in men (adjusted OR [AOR] = 8.57, 95% CI = 3.79–19.41). Likewise, among women, a higher value of motherhood was associated with a higher likelihood of willingness for childbirth (AOR = 10.42, 95% CI = 3.65–29.80). Additionally, in women, a higher monthly allowance was associated with a higher likelihood of willingness for childbirth in women (AOR = 1.02, 95% CI = 1.01–1.03) (Table 4).

**Table 4:** Factors influencing willingness for childbirth among men and women among college students from South Korea in 2021: logistic regression (N = 286)

Variables	Categories		Men (n = 144)				Women (n = 142)	
		Adj. OR	95% Cl	*p*		Adj. OR	95% Cl	*p*
Allowance		1.01	1.00-1.03	0.094		1.02	1.01–1.03	0.002
Religious (Ref. no)	Yes	1.89	0.67–5.30	0.227		2.26	0.97–5.28	0.059
Value of motherhood or fatherhood (Ref. low score; (8–25) ^a^ / (8–22)^b^)	High score; (26–32) ^a^ / (23–32) ^b^	8.57	3.79–19.41	< 0.001		10.42	3.65–29.80	< 0.001

^a^ Based on mean scores of total values for men; ^b^ Based on mean scores of total values for women; *Adj. OR* adjusted odds ratio; *CI* confidence interval; *Ref* Reference

## Discussion

This study identified gender differences in willingness for childbirth, fertility knowledge, and value of motherhood or fatherhood among college students, and both similarities and differences in factors influencing willingness for childbirth were found between men and women.

First, female students in this study showed lower willingness for future childbirth than male students; this finding is consistent with the recent national health and sociological studies on young adults reporting that women had more negative attitudes towards having children than men [20, 21]. A reason why women avoid childbirth may be that as the number of employed women increases, women have a greater burden due to childbirth and childrearing than men in the context of balancing work and childcare. Another explanatory factor may be the deeply rooted Confucian culture in Korea, which regards procreation as preserving and extending the lineage for men [7, 22].

In addition, with regard to value of motherhood or fatherhood, women valued motherhood significantly less than men valued fatherhood. Similarly, another study conducted in Korea [7] found that women perceived parenthood as having a more negative impact on their careers and lives and placed a lower value on having children than men. In the traditional culture of Korea, following Confucian gender roles, women are considered the primary caregivers of their children [22]. Furthermore, while women’s social activities have increased, men’s roles and duties in housekeeping have not substantially changed [20, 22]. Previous studies conducted among college students in Korea have also shown that, although the value of marriage has changed over time, the roles of women and men are still divided according to patriarchal values, and gender inequality still exists [7, 23]. This could possibly explain why women placed a lower value on motherhood. Therefore, this explanation suggests that it is necessary to develop policies or provide adequate resources for women so that parenthood does not interfere with women’s careers. From a societal perspective, it is also necessary to change perceptions of the roles of motherhood and fatherhood in the family.

Women had a lower fertility knowledge score than men in this study, which is similar to the results of previous studies conducted among university students in Korea using the same fertility knowledge instrument [9], while another study conducted among individuals of childbearing age in 74 countries found women had higher knowledge scores than men [18]. Since research findings are inconsistent in the literature, further research is needed to validate these gender differences in fertility knowledge. Meanwhile, a lack of fertility knowledge in young adults, regardless of childbirth plans, could lead to childlessness in the near future [11]. Therefore, it is necessary to provide concrete education on fertility issues in schools and health clinics in the community to help young adults in Korea make informed reproductive decisions.

In the present study, religion was found to show a significant association with future willingness for childbirth in both men and women. These findings are in line with the results of previous studies that have consistently shown religion to be an important predictor of fertility intentions and behaviors; specifically, religious people have been found to have both more intentions and more children than non-religious people in many countries [14, 24]. Mencarini, Vignoli, and Gottard [25] explained that religious people are more likely to express willingness for childbirth because their attitudes toward children are more positive. In addition, religious networks may supply social support and encouragement regarding religious values, and subjective social normative pressure for childbirth may be more relevant for religious individuals [26, 27]. These results suggest that religiosity still seems to have a positive effect on perceptions of parenthood even in the current modern era.

Furthermore, this study confirmed that the value of motherhood or fatherhood was an important factor influencing willingness for childbirth in the future in both men and women. Moreover, according to the National Health Study conducted in Korea, which tracked whether values toward parenthood influenced childbirth using cross-sectional data obtained at different times from married women, these values affect not only willingness for childbirth but also actual childbearing behavior [20]. Therefore, social effort may be required to provide supportive resources so that being a parent can be as favorable of an experience as possible for parents to enjoy their lives. Currently, the Korean government has established policies to encourage childbirth, such as providing financial support for infertile couples to undergo artificial reproductive technology, antenatal care, and childcare [28]. However, we need to bridge the gap between the current policy and practical needs by confirming the degree to which these policies are actually helpful to couples. The finding that the value of motherhood or fatherhood had a significant influence on both men and women on the willingness for future childbirth suggests useful evidence and directions for future preconception care and education for young adults in order to encourage them to plan future pregnancies in advance for healthy pregnancy and childbirth.

The present study found that the monthly allowance significantly influenced female students’ willingness for childbirth in the future, which is in line with previous study findings that socioeconomic status is positively associated with higher future childbearing intention for women in many developed countries [14, 29]. A potential explanation for this may be that in countries where women reach a higher economic status and have higher education, other social structural circumstances that can affect fertility help women combine work with having children [14]. On the contrary, women in developing countries are likely to be more willing to give birth because their educational and economic levels are much lower, which might be explained by the expected benefits of children’s work in agricultural-centered societies [30]. In this study, female students’ allowances were not a direct indicator of economic status (such as income, education, or employment), but this variable can be interpreted as a reflection of the financial situation practically experienced by female students.

In this study, health behavior characteristics were not associated with willingness for future childbirth in both men and women. Few studies have evaluated the association between willingness for childbirth and health behavior in young adults; therefore, it is difficult to make a clear comparison. However, previous studies confirmed that fertility knowledge was related to lifestyle habits (e.g., smoking and alcohol consumption) and sexual behavior (e.g., sexually transmitted infections) [31, 32]. In addition, women who currently smoked and used alcohol had a higher risk of unplanned pregnancy [15]. Further research is necessary to explore the association between fertility intention and health behavior in young adults.

The desired number of children in this study was about 2.1, which is consistent with the recent Korean national health study on young adults [33]. However, the total fertility rate was 0.78 children per woman in 2022, which declined from 0.81 to 2021 in South Korea [1]. These differences between the desired number of children and actual birth could be explained by culturally shared subjective norms, such as the presence of cultural schema about two children in Korea, yet socioeconomic conditions and government policy could be a barrier to actual childbirth [33].

Our study has several limitations. First of all, since this study was conducted through an online survey for the convenience of recruitment, caution is needed in the interpretation of the findings due to the possibility that the reliability and validity of the research data may be diminished by careless and random responses on an online survey. Second, since this study asked about participants’ thoughts about childbirth in the future, not the same period when the study was conducted, participants’ responses may not necessarily be consistent with their real thoughts about childbirth in the future. Third, our study issued recruiting flyers through campus e-mails from one university in Korea, even though participants were recruited simultaneously through other online communities for other college students, which could be a source of selection bias. In addition, the findings are not generalizable to all young adults since we conducted this study among college students. Future research needs to investigate larger study groups of young adults. Finally, our findings need further investigation after proven validity and reliability further development of the instrument in these areas because the reliability (using Cronbach’s alpha) of some instruments remained under 0.7, which could affect the study findings.

Despite the limitations of the study, it makes a significant contribution by focusing on gender differences in the future childbirth willingness of college students, as well as by identifying novel associations with future childbirth willingness and the value of parenthood. In addition, the findings of this study provide insight into factors influencing future childbirth willingness in men and women. From the perspective of RLP, this study is meaningful since investigating willingness and thoughts about future childbirth among college students provides an opportunity to consider planning for childbearing in advance, which will promote healthy pregnancy and childbirth in the future.

## Conclusion

The findings of this study indicate that there were differences in willingness for childbirth, fertility knowledge, and value of motherhood or fatherhood according to gender in college students. The value of motherhood or fatherhood was an important influencing factor on the willingness for childbirth in both male and female college students, whereas the monthly allowance was also an important influencing factor in female college students. Based on these findings, we suggest the following for future research and practice. First of all, future research needs to explore the association between willingness for childbirth and the value of motherhood or fatherhood in larger study groups of young adults, and potential connections with perceptions of gender roles within the household might be further addressed. Another direction for future research could be to explore the intention-behavior connection by applying the framework of the theory of planned behavior (TPB) to the study. Furthermore, the results of this study indicate that future research should develop effective interventions for informed reproductive decision-making for college students considering gender differences.

## Data Availability

The datasets generated during and/or analyzed during the current study are not publicly available due to protection and confidentiality but may be available from the corresponding author upon reasonable request.

## List of Abbreviations

Korea: South Korea
OECD: Organisation for Economic Co-operation and Development
The CDC: Center for Disease Control and prevention
RLP: Reproductive Life Planning
OR: Odd Ratio
IRB: the Institutional Review Board
No: Number
SPSS: Statistical Package for Social Sciences
IBM: International Business Machines corporation
NY: New York
USA: United States of America
AOR: Adjusted OR
CI: Confidence interval
TPB: Theory of planned behavior
